# Cervical Cancer Screening via Visual Inspection With Acetic Acid and Lugol Iodine for Triage of HPV-Positive Women

**DOI:** 10.1001/jamanetworkopen.2024.4090

**Published:** 2024-03-29

**Authors:** Sumeng Wang, Le Dang, Shujun Liu, Remila Rezhake, Huijiao Yan, Xianzhi Duan, Le Zhang, Linlin Zhang, Lifeng Zhang, Meili Su, Fumei Guo, Cailing Yan, Meili Liu, Xiaoyan Cao, Min Sun, Youlin Qiao, Fanghui Zhao

**Affiliations:** 1 National Cancer Center/National Clinical Research Center for Cancer/Cancer Hospital, Chinese Academy of Medical Sciences and Peking Union Medical College, Beijing, China; 2 Peking Union Medical College Hospital, Chinese Academy of Medical Sciences and Peking Union Medical College, Beijing, China; 3 Cancer Research Institute, Affiliated Cancer Hospital of Xinjiang Medical University, Urumqi, China; 4 Department of Global Health, School of Population Medicine and Public Health, Chinese Academy of Medical Sciences and Peking Union Medical College, Beijing, China; 5 Department of Obstetrics and Gynecology, Beijing Tongren Hospital Affiliated to Capital Medical University, Beijing, China; 6 Dean’s Office, Ordos Maternal and Child Health Care Hospital, Inner Mongolia Autonomous Region, Ordos, China; 7 Women’s Health Department, Ikinholo Banner Maternal and Child Care Center, Ordos City, Inner Mongolia Autonomous Region, Ordos, China; 8 Women’s Health Department, Otog Front Banner Maternal and Child Care Center, Ordos City, Inner Mongolia Autonomous Region, Ordos, China; 9 Dean’s Office, Dalad Banner Maternal and Child Care Center, Ordos City, Inner Mongolia Autonomous Region, Ordos, China; 10 Women’s Health Department, Jungar Banner Maternal and Child Care Center, Ordos City, Inner Mongolia Autonomous Region, Ordos, China; 11 Women’s Health Department, Otog Banner Maternal and Child Care Center, Ordos City, Inner Mongolia Autonomous Region, Ordos, China; 12 Department of Obstetrics and Gynecology, Hangjin Banner Maternal and Child Care Center, Ordos City, Inner Mongolia Autonomous Region, Ordos, China; 13 Women’s Health Department, Uxin Banner Maternal and Child Care Center, Ordos City, Inner Mongolia Autonomous Region, Ordos, China; 14 Department of Obstetrics and Gynecology, Dongsheng District Maternal and Child Care Center, Ordos City, Inner Mongolia Autonomous Region, Ordos, China

## Abstract

**Importance:**

Limited evidence supports the performance of human papillomavirus (HPV) DNA testing as a primary screening method, followed by triage with visual inspection with acetic acid, in areas with limited health care resources, as suggested by the 2021 World Health Organization guidelines.

**Objective:**

To evaluate the performance of visual inspection with acetic acid and Lugol iodine as a triage method for detecting cervical precancerous lesions among HPV-positive women in 1 visit.

**Design, Setting, and Participants:**

This cohort study examined the implementation of a government-led cervical cancer screening program conducted from January 1, 2016, to December 31, 2020, in Ordos City, China. Female residents, aged 35 to 64 years, who understood the screening procedures and voluntarily participated were included in the study. Women were excluded if they reported never having had sexual intercourse, were pregnant, had a hysterectomy, or had ever undergone treatment for cervical lesions. Statistical analysis was conducted from December 2022 to December 2023.

**Exposures:**

The program used the careHPV DNA assay as the primary screening method, and immediate triage was performed by visual inspection if HPV screening results were positive, with a 5-year screening interval. A colposcopy was performed for the women who had suspected cancer on visual inspection results or who were HPV positive and had abnormal visual inspection results, all in 1 visit.

**Main Outcomes and Measures:**

The rate of compliance with colposcopy and the detection rate of cervical intraepithelial neoplasia grade 2 or higher (CIN2+).

**Results:**

The study included 187 863 women (median age, 46 years [IQR, 40-52 years]) who participated in the program and had valid HPV test results. The overall prevalence of HPV positivity was 12.8% (24 070 of 187 863), and the adherence to triage with visual inspection among HPV-positive women was 93.9% (22 592 of 24 070). Among HPV-positive women, the rate of compliance with colposcopy was 65.6% (2714 of 4137), and the CIN2+ detection rate was 2.8% (643 of 22 592).

**Conclusions and Relevance:**

The findings of this cohort study suggest that the implementation of HPV testing, visual inspection, and colposcopy within 1 visit may mitigate losses to follow-up, detect precancerous lesions, and hold significant implications for screening in comparable areas with limited health care resources.

## Key Points

**Question:**

Is it feasible to implement human papillomavirus (HPV) primary screening with visual inspection with acetic acid and Lugol iodine triage in areas with limited health care resources?

**Findings:**

In this cohort study of 186 863 women in China, an HPV DNA assay was used for primary screening, and visual inspections were used for immediate triage; if both were positive, a referral for colposcopy was conducted within 1 visit. There was a 93.9% completion rate for the triage test and a detection rate of 2.8% for cervical intraepithelial neoplasia grade 2 or higher among HPV-positive individuals.

**Meaning:**

This study suggests that the implementation of an HPV DNA assay with visual inspection and a colposcopy within 1 visit may mitigate losses to follow-up, detect precancerous lesions, and hold promise for use in low-resource settings.

## Teaser

This cohort study evaluates the performance of visual inspection with acetic acid and Lugol iodine as a triage method for detecting cervical precancerous lesions among human papillomavirus (HPV)–positive women in 1 visit.

## Introduction

Cervical cancer has posed a heavy disease burden in low- and middle-income countries, where approximately 90% of cases of cervical cancer occur.^1^ In China, an estimated 150 700 new cases and 55 700 deaths were attributed to cervical cancer in 2022,^2^ with the peak age of cervical cancer incidence occurring between 50 and 54 years.^3^ The World Health Organization (WHO) proposed screening 70% of women with a high-quality test as a crucial intervention in the pursuit of eliminating cervical cancer.^4^ Although screening has shown significant success in high-income countries throughout the past few decades,^5,6^ the implementation of similar screening programs has faced significant challenges in resource-constrained settings.^7^

Cytology has historically been the standard primary screening method and has played a crucial role in reducing cervical cancer mortality. Conversely, in low-resource settings, challenges in promoting cytology stem largely from the limited availability of cytology experts, the high costs, and the need for multistep visits, as required by the cytology-based screening algorithm.^8^ In 2021, the WHO recommended human papillomavirus (HPV) DNA–based tests as the preferred primary screening approach,^9^ which has gained wide acceptance and has robust diagnostic accuracy even in low-resource areas.^10^

Most HPV infections clear spontaneously within a span of 12 to 24 months. As a result, it is essential to perform triage among HPV-positive individuals and refer them to colposcopy for further diagnosis.^11^ Triage among HPV-positive individuals helps prevent overdiagnosis and unnecessary overtreatment. However, the challenges posed by the high cost, limited availability of adequately trained physicians, and poor adherence to further diagnosis among HPV-positive women in low-resource areas are of concern. As the fifth of 7 algorithms, the WHO recommended visual inspection with acetic acid as an alternative triage method for HPV-positive women to be implemented in low-resource settings where cytology is constrained.^9^ Visual inspection with acetic acid offers several advantages, including affordability, ease of use, immediate results, and reduced need for recall rounds. Currently, the performance of visual inspection with acetic acid triage among large samples of HPV-positive women has limited supporting evidence.^12,13^

In 2009, a government-led pilot program for cervical cancer screening was initiated in 221 counties in rural China. The program was included in the basic public health services project and expanded to cover the entire female population aged 35 to 64 years in 2019.^14^ Currently, only 36.8% of women aged 35 to 64 years have ever undergone cervical cancer screening.^15^ Despite being an upper- and middle-income country, China’s challenges in health care access arise due to its large population, uneven development across the central and western regions, and disparities in the distribution of medical resources. As a consequence, accessing medical care becomes difficult for individuals living in areas with limited health resources. This article aims to assess the performance of HPV testing as a primary screening method followed by triage with visual inspection with acetic acid and Lugol iodine for detecting cervical precancerous lesions among women in regions of China with limited health care resources. In addition, the study seeks to use the findings from Ordos City to provide practical insights for other regions facing similar challenges in terms of limited health care resources.

## Methods

### Study Setting, Population, and Design

Ordos City, a vast territory with a sparsely distributed, ethnically diverse population, has experienced a high prevalence of cervical cancer over the years. Despite being an economically affluent region in China, Ordos City faced challenges in terms of limited access to health care services, particularly scarcity of highly qualified physicians.^16^ This study was granted an ethical exemption by the institutional review board of the National Cancer Center/Cancer Hospital, Chinese Academy of Medical Sciences because the data were deidentified. Participants provided written informed consent. The study followed the Strengthening the Reporting of Observational Studies in Epidemiology (STROBE) reporting guideline.

The government-led cervical cancer screening program was conducted from January 1, 2016, to December 31, 2020. The program aimed to reach 60% of women aged 35 to 64 years residing in Ordos City, which equates to approximately 203 400 women.^17^ Considering previous surveys and internal data in Ordos City, the organizational screening coverage was set at 60% based on the assumption that 10% of women would participate in opportunistic screening, resulting in an overall screening rate of 70%, as proposed by the WHO. Free screening services were offered at maternal and child care centers in 9 districts or banners. The eligibility of the screening included female residents, aged 35 to 64 years, who understood the screening procedures and voluntarily participated. Women were excluded if they reported never having had sexual intercourse, were pregnant, had a hysterectomy, or had ever undergone prior treatment for cervical lesions.

### Screening Procedures

Local health workers and village physicians reached out to eligible women in advance. Subsequently, women independently visited the screening site after receiving a notification. At each screening site, health workers provided a brief education session, and sociodemographic information was collected. Subsequently, health workers confirmed that the participants were not menstruating, had not received vaginal administration of medication or vaginal irrigation, and had abstained from sexual activity in the preceding 48 hours. Women underwent a general gynecologic examination in which HPV specimens were collected, followed by a simultaneous visual inspection. The Figure displays the flowchart of the organized screening program.

**Figure.**
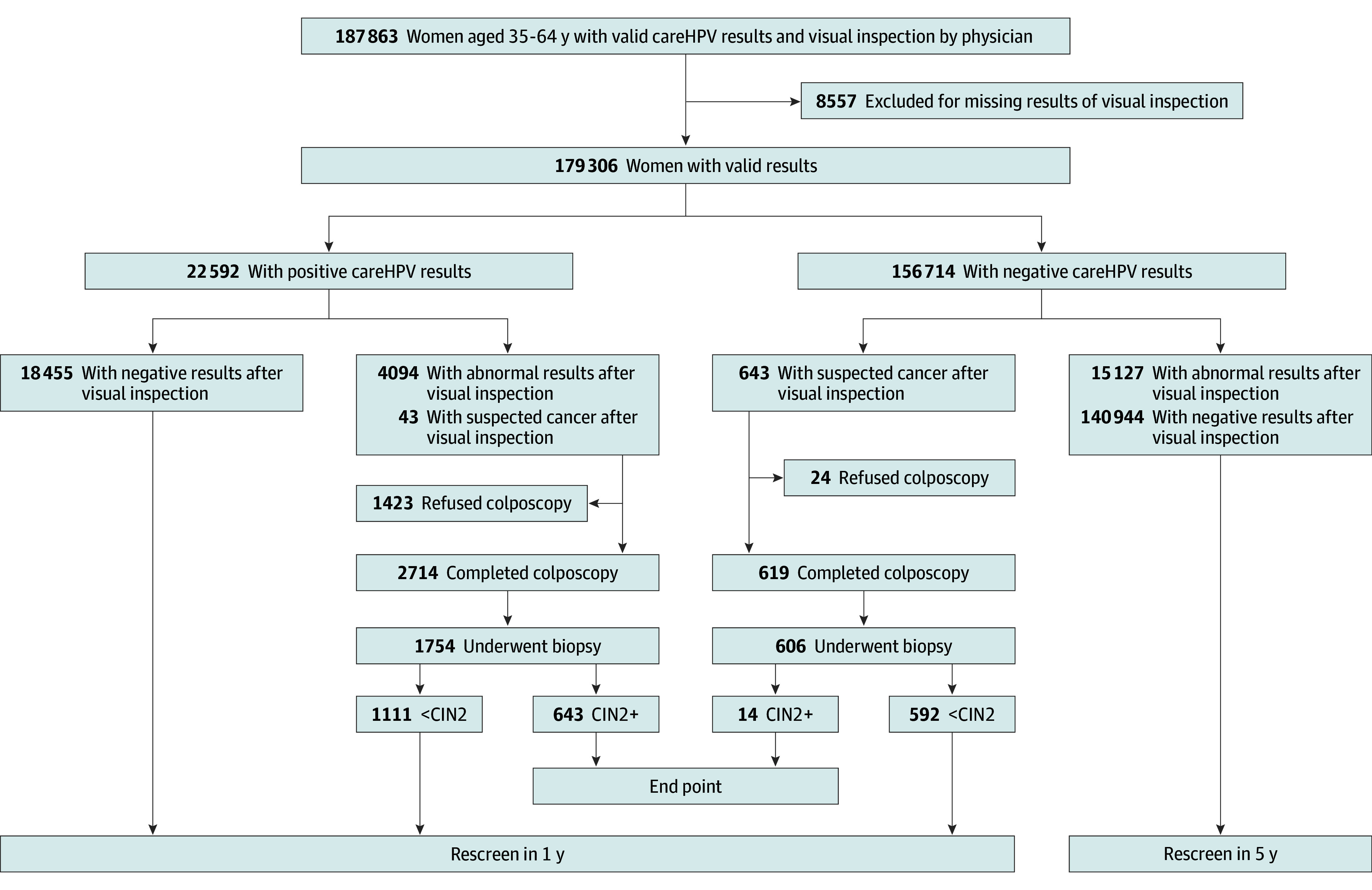
Flowchart of the Screening Program and Eligible Women in the Study CIN2 indicates cervical intraepithelial neoplasia grade 2; CIN2+, cervical intraepithelial neoplasia grade 2 or higher.

### Screening Tests

Human papillomavirus sampling was performed for each woman. The program used the WHO prequalified careHPV test (Qiagen) as the primary screening method.^18^ The careHPV test is a simplified and rapid HPV DNA assay specifically designed for screening women in low-resource regions. The assay detects a pool of 14 high-risk HPV types (HPV-16, -18, -31, -33, -35, -39, -45, -51, -52, -56, -58, -59, -66, and -68).

The careHPV test equipment requires only a small area of workspace. It does not require electricity or running water and can be performed rapidly with minimal training required. Therefore, local laboratory technicians in Ordos City conducted careHPV testing at the screening sites in each district or banner. The test is capable of producing accurate results within 2.5 hours. The results of the careHPV testing were HPV negative or HPV positive.

Visual inspection was performed in parallel with the HPV test as cotesting for each woman. Visual inspection with acetic acid and Lugol iodine involves the examination of the uterine cervix with the naked eye after the application of acetic acid and Lugol iodine, 3% to 5%, which provides simple tests for the early detection of cervical precancerous lesions. The examination does not require any laboratory support, and its result is immediately available. A visual inspection with acetic acid with abnormal results was defined as the observation of thickened white plaques or acetowhite areas usually near the squamocolumnar junction of the cervix. These areas become visible at least 1 minute after the application of acetic acid. A visual inspection with Lugol iodine with abnormal results was defined as bright yellow or mustard yellow areas of the same zone on the cervix, visible after the application of Lugol iodine. If the detected lesion exhibited a cauliflower-like (fungating) mass or ulcer, it was considered suspicious for cancer. The results of the visual inspection were negative, abnormal, or suspected cancer.^9^

### Colposcopy, Biopsy, and Follow-Up

Visual inspection served as an immediate triage method for HPV-positive women. Women with abnormal visual inspection results were asked to wait half a day for the HPV test results. If women were HPV positive, a colposcopy was conducted during the same visit. On the other hand, women who were diagnosed as HPV positive but who had negative visual inspection results were recalled for rescreening in the subsequent year. Within the framework of this cotesting design, the immediate colposcopy was performed only when women received a diagnosis of suspected cancer on visual inspection, regardless of the HPV test results.

Digital colposcopes (Zonsun Healthcare Co Ltd) were used at each screening site. If the colposcopy results were normal, no biopsy was performed. In cases in which an abnormal colposcopy result occurred and potential lesions were identified, local colposcopists conducted biopsies. The pathology results, which were considered the criterion standard, were classified into different categories: normal or inflammatory, cervical intraepithelial neoplasia 1 (CIN1), CIN2, CIN3, and carcinoma. According to current guidelines in China, women who had CIN2 or higher (CIN2+) confirmed on biopsy were referred to clinical treatment. Women with pathologic findings less than CIN2 were scheduled for a 1-year follow-up. Women who were HPV negative and had normal or abnormal visual inspection results at baseline underwent a 5-year follow-up.

### Quality Control and Data Management

Local health care workers were authorized to be involved in our program only if they passed the training before the program started. A database for a regional cervical cancer screening information system was established to gather information from the women who underwent screening, while avoiding duplicate screenings. Data extraction for this study was conducted from the electronic database. The extracted data encompassed women’s demographic information, clinical diagnoses from each visit, HPV laboratory test results, and pathology diagnoses.

### Statistical Analysis

Statistical analysis was conducted from December 2022 to December 2023. The primary outcomes were the rate of compliance with colposcopy and the detection rate of CIN2+. The parameters listed were calculated and stratified by a 5-year increment age group: (1) number of women screened; (2) number of colposcopies; (3) colposcopy compliance rate; (4) number of histologically confirmed cases of CIN2+ and CIN3+; (5) positive predictive value (PPV) for CIN2+ and CIN3+; (6) number needed to refer to colposcopy to detect 1 case of CIN2+ (or CIN3+): number of women who underwent colposcopies/number of CIN2+ (or CIN3+) identified; and (7) pathologic findings.

The demographic characteristics of the participants were summarized using frequencies and percentages. Differences in outcome distribution among different testing results or age groups were tested using the χ^2^ test. All analyses were conducted using SAS, version 9.4 (SAS Institute Inc). All *P* values were from 2-sided tests and results were deemed statistically significant at *P* < .05.

## Results

### Characteristics of the Study Population

Of the 187 863 women (median age, 46 years [IQR, 40-52 years]) who participated in the screening program and had valid HPV results, 24 070 were found to be HPV positive, resulting in an overall HPV positivity rate of 12.8% (Figure). Among the participants, 8557 women refused to undergo visual inspection (1478 HPV positive and 7079 HPV negative). The overall adherence to triage with visual inspection among HPV-positive women was 93.9% (22 592 of 24 070).

Table 1 displays the baseline demographic characteristics of the 179 306 women who had valid results for HPV testing and visual inspection in the study. The median age of this group was 46 years (IQR, 40-52 years). Overall, most women had attained at least a high school degree (60.0% [107 548]), 39 245 (21.9%) were classified as being in menopausal status (median age of menopausal women, 50 years [IQR, 48-53 years]), and most women (60.5% [108 561]) reported not using contraceptives.

**Table 1.** Characteristics of Women With Valid Results

Characteristic^a^	Women, No. (%)		
	HPV positive	HPV negative	Total
Total	22 592 (12.6)	156 714 (87.4)	179 306 (100.0)
Age group, y			
Median (IQR)	46 (40-53)	45 (40-52)	46 (40-52)
35-44	9777 (43.3)	71 266 (45.5)	81 043 (45.2)
45-54	8022 (35.5)	57 983 (37.0)	66 005 (36.8)
55-64	4793 (21.2)	27 465 (17.5)	32 258 (18.0)
History of screening			
Screened before	8736 (38.7)	65 200 (41.6)	73 936 (41.2)
Never screened	5685 (25.2)	38 138 (24.3)	43 823 (24.4)
Unknown	8171 (36.2)	53 376 (34.1)	61 547 (34.3)
Ethnicity			
Han	20 641 (91.4)	145 586 (92.9)	166 227 (92.7)
Minority	1888 (8.4)	10 703 (6.8)	12 591 (7.0)
Missing	63 (0.3)	425 (0.3)	488 (0.3)
Marital status			
Married	21 771 (96.4)	152 681 (97.4)	174 452 (97.3)
Single	192 (0.8)	1522 (1.0)	1714 (1.0)
Other	558 (2.5)	2056 (1.3)	2614 (1.5)
Missing	71 (0.3)	455 (0.3)	526 (0.3)
Educational level			
Primary school or below	9147 (40.5)	62 092 (39.6)	71 239 (39.7)
High school	10 661 (47.2)	74 104 (47.3)	84 765 (47.3)
University or other degree	2714 (12.0)	20 069 (12.8)	22 783 (12.7)
Missing	70 (0.3)	449 (0.3)	519 (0.3)
Insurance			
No	833 (3.7)	6595 (4.2)	7428 (4.1)
Yes	21 615 (95.7)	149 009 (95.1)	170 624 (95.2)
Missing	144 (0.6)	1110 (0.7)	1254 (0.7)
Menopause			
No	16 335 (72.9)	122 464 (78.7)	138 799 (77.4)
Yes	6081 (27.1)	33 164 (21.3)	39 245 (21.9)
Missing	176 (0.8)	1086 (0.7)	1262 (0.7)
Marriage age, y			
<23	11 678 (51.7)	76 151 (48.6)	87 829 (49.0)
≥23	10 777 (47.7)	79 784 (50.9)	90 561 (50.5)
Missing	137 (0.6)	779 (0.5)	916 (0.5)
No. of sexual partners			
≤1	22 260 (98.5)	154 806 (98.8)	177 066 (98.8)
≥2	234 (1.0)	1293 (0.8)	1527 (0.9)
Missing	98 (0.4)	615 (0.4)	713 (0.4)
Postcoital bleeding			
Yes	928 (4.1)	5419 (3.5)	6347 (3.5)
No	21 526 (95.9)	150 431 (96.0)	171 957 (95.9)
Missing	138 (0.6)	864 (0.6)	1002 (0.6)
Abnormal vaginal discharge			
Yes	5286 (23.4)	35 307 (22.5)	40 593 (22.6)
No	17 161 (76.0)	120 482 (76.9)	137 643 (76.8)
Missing	145 (0.6)	925 (0.6)	1070 (0.6)
Contraceptive use			
Yes	8809 (39.0)	58 146 (37.1)	66 955 (37.3)
No	13 241 (58.6)	95 320 (60.8)	108 561 (60.5)
Unknown	542 (2.4)	3248 (2.1)	3790 (2.1)

Abbreviation: HPV, human papillomavirus.

^a^ All *P* < .05.

### Overall Screening Findings

Of 22 592 women who tested positive for HPV with valid visual inspection results, 4094 (18.1%) had abnormal visual inspection results, and 43 (0.2%) had suspected cancer based on visual inspection results. Meanwhile, of 156 714 women who tested negative for HPV, 643 (0.4%) had suspected cancer based on visual inspection. In total, 30.3% of women (1447 of 4780) refused colposcopy. Among the 4780 women referred to colposcopy, immediate referrals were provided for 686 women with suspected cancer based on visual inspection. The compliance rate for these immediate referrals was high (95.0% [652 of 686]).

Among women who tested positive for HPV, visual inspection reduced colposcopy referral rates to 18.3% (4137 of 22 592) (Table 2). The overall rate of compliance with colposcopy was 65.6% (2714 of 4137). The colposcopy compliance rate was 65.5% (2681 of 4094) among those with abnormal visual inspection results and 76.7% (33 of 43) among those with suspected cancer based on visual inspection.

**Table 2.** Screening Findings Among Women Who Had careHPV-Positive and Abnormal Results or Suspected Cancer Based on Visual Inspection, Stratified by Age

Visual inspection results, stratified by age	No. screened	No. who underwent colposcopy (%)	Colposcopy compliance rate, %	Cases of CIN2+ detected, No. (%)	PPV for CIN2+, % (95% CI)	NNR to colposcopy to detect 1 case of CIN2+	Cases of CIN3+ detected, No. (%)	PPV for CIN3+, % (95% CI)	NNR to colposcopy to detect 1 case of CIN3+
**Women aged 35-39 y**									
Abnormal	1169	728 (98.9)	62.3	192 (99.0)	26.4 (23.2-29.7)	3.8	126 (99.2)	17.3 (14.6-20.3)	5.8
Suspected cancer	10	8 (1.1)	80.0	2 (1.0)	25.0 (3.2-65.1)	4.0	1 (0.8)	12.5 (0.3-52.7)	8.0
Subtotal	1179	736 (27.1)	62.4	194 (30.2)	26.4 (23.2-29.7)	3.8	127 (29.0)	17.3 (14.6-20.2)	5.8
**Women aged 40-44 y**									
Abnormal	990	639 (99.1)	64.6	172 (97.7)	26.9 (23.5-30.5)	3.7	115 (97.5)	18.0 (15.1-21.2)	5.6
Suspected cancer	6	6 (0.9)	100.0	4 (2.3)	66.7 (22.3-95.7)	1.5	3 (2.5)	50.0 (11.8-88.2)	2.0
Subtotal	996	645 (23.8)	64.8	176 (27.4)	27.3 (23.9-30.9)	3.7	118 (26.9)	18.3 (15.4-21.5)	5.5
**Women aged 45-49 y**									
Abnormal	792	516 (98.3)	65.2	112 (94.1)	21.7 (18.2-25.5)	4.6	78 (92.9)	15.1 (12.1-18.5)	6.6
Suspected cancer	12	9 (1.7)	75.0	7 (5.9)	77.8 (40.0-97.2)	1.3	6 (7.1)	66.7 (29.9-92.5)	1.5
Subtotal	804	525 (19.3)	65.3	119 (18.5)	22.7 (19.2-26.5)	4.4	84 (19.2)	16.0 (13.0-19.4)	6.3
**Women aged 50-54 y**									
Abnormal	518	356 (98.3)	68.7	61 (92.4)	17.1 (13.4-21.5)	5.8	42 (89.4)	11.8 (8.6-15.6)	8.5
Suspected cancer	8	6 (1.7)	75.0	5 (7.6)	83.3 (35.9-99.6)	1.2	5 (10.6)	83.3 (35.9-99.6)	1.2
Subtotal	526	362 (13.3)	68.8	66 (10.3)	18.2 (14.4-22.6)	5.5	47 (10.7)	13.0 (9.7-16.9)	7.7
**Women aged 55-59 y**									
Abnormal	394	281 (99.7)	71.3	48 (98.0)	17.1 (12.9-22.0)	5.9	30 (96.8)	10.7 (7.3-14.9)	9.4
Suspected cancer	1	1 (0.4)	100.0	1 (2.0)	100.0 (2.5-100.0)	1.0	1 (3.2)	100.0 (2.5-100.0)	1.0
Subtotal	395	282 (10.4)	71.4	49 (7.6)	17.4 (13.1-22.3)	5.8	31 (7.1)	11.0 (7.6-15.2)	9.1
**Women aged 60-64 y**									
Abnormal	231	161 (98.2)	69.7	36 (92.3)	22.4 (16.2-29.6)	4.5	28 (90.3)	17.4 (11.9-24.1)	5.8
Suspected cancer	6	3 (1.8)	50.0	3 (7.7)	100.0 (29.2-100.0)	1.0	3 (9.7)	100.0 (29.2-100.0)	1.0
Subtotal	237	164 (6.0)	69.2	39 (6.1)	23.8 (17.5-31.0)	4.2	31 (7.1)	18.9 (14.3-17.1)	5.3
**Total**									
Abnormal	4094	2681 (98.8)	65.5	621 (96.6)	23.2 (21.6-24.8)	4.3	419 (95.7)	15.6 (13.2-25.7)	6.4
Suspected cancer	43	33 (1.2)	76.7	22 (3.4)	66.7 (48.2-82.0)	1.5	19 (4.3)	57.6 (39.2-74.5)	1.7
Total	4137	2714	65.6	643 (2.8)	23.7 (22.1-25.3)	4.2	438 (1.9)	16.1 (14.8-17.6)	6.2

Abbreviations: CIN2+, cervical intraepithelial neoplasia grade 2 or higher; CIN3+, cervical intraepithelial neoplasia grade 3 or higher; NNR, number needed to refer; PPV, positive predictive value.

The detection rates were 2.8% (643 of 22 592) for CIN2+ and 1.9% (438 of 22 592) for CIN3+. Triage with visual inspection exhibited a higher detection rate of CIN2+ and CIN3+ among younger women (CIN2+: χ^2^_1_ = 20.0034; *P* = .001; CIN3+: χ^2^_2_ = 12.0065; *P* = .04). Among women who had abnormal or suspected cancer based on visual inspection, the PPV for CIN2+ was 23.7% (95% CI, 22.1%-25.3%), and the PPV for CIN3+ was 16.1% (95% CI, 14.8%-17.6%) (Table 2).

### Screening Findings Stratified by Age

Table 3 presents the age-stratified pathologic findings among women who tested positive for HPV, with abnormal results or suspected cancer based on visual inspection. Table 4 displays the screening findings among age-stratified, HPV-negative women, with suspected cancer results based on visual inspection. A total of 5 cases of cancer were detected. For women who tested positive for HPV and had abnormal results or suspected cancer based on visual inspection, the detection rate of CIN2+ was significantly higher compared with women who were HPV negative with suspected cancer based on visual inspection (15.5% [643 of 4137] vs 2.2% [14 of 643]; χ^2^ = 83.8509; *P* < .001).

**Table 3.** Pathologic Findings Among Women With careHPV-Positive and Abnormal Results or Suspected Cancer Based on Visual Inspection, Stratified by Age

Visual inspection results, stratified by age	No. (%)				
	Normal	CIN1	CIN2	CIN3	Cancer
**Women aged 35-39 y**					
Abnormal	176 (97.2)	107 (99.1)	66 (98.5)	117 (100.0)	9 (90.0)
Suspected cancer	5 (2.8)	1 (0.9)	1 (1.5)	0	1 (10.0)
Subtotal	181 (24.8)	108 (28.4)	67 (32.7)	117 (30.4)	10 (18.9)
**Women aged 40-44 y**					
Abnormal	168 (98.8)	98 (100.0)	57 (98.3)	113 (98.3)	2 (66.7)
Suspected cancer	2 (1.2)	0	1 (1.7)	2 (1.7)	1 (33.3)
Subtotal	170 (23.3)	98 (25.7)	58 (28.3)	115 (29.9)	3 (5.7)
**Women aged 45-49 y**					
Abnormal	154 (99.4)	65 (100.0)	34 (97.1)	69 (98.6)	9 (64.3)
Suspected cancer	1 (0.7)	0	1 (2.9)	1 (1.4)	5 (35.7)
Subtotal	155 (21.2)	65 (17.1)	35 (17.1)	70 (18.2)	14 (26.4)
**Women aged 50-54 y**					
Abnormal	100 (99.0)	50 (100.0)	19 (100.0)	33 (94.3)	9 (75.0)
Suspected cancer	1 (1.0)	0	0	2 (5.7)	3 (25.0)
Subtotal	101 (13.8)	50 (13.1)	19 (9.3)	35 (9.1)	12 (22.6)
**Women aged 55-59 y**					
Abnormal	81 (100.0)	33 (100.0)	18 (100.0)	27 (100.0)	3 (75.0)
Suspected cancer	0	0	0	0	1 (25.0)
Subtotal	81 (11.1)	33 (8.7)	18 (8.8)	27 (7.0)	4 (7.5)
**Women aged 60-64 y**					
Abnormal	42 (100.0)	27 (100.0)	8 (100.0)	20 (95.2)	8 (80.0)
Suspected cancer	0	0	0	1 (4.8)	2 (20.0)
Subtotal	42 (5.8)	27 (7.1)	8 (3.9)	21 (5.6)	10 (18.9)
**Total**					
Abnormal	721 (98.8)	380 (99.7)	202 (98.5)	379 (98.4)	40 (75.5)
Suspected cancer	9 (1.2)	1 (0.3)	3 (1.5)	6 (1.6)	13 (24.5)
Total	730	381	205	385	53

Abbreviations: CIN1, cervical intraepithelial neoplasia grade 1; CIN2, cervical intraepithelial neoplasia grade 2; CIN3, cervical intraepithelial neoplasia grade 3.

**Table 4.** Screening Findings Among Women With careHPV-Negative Results and Suspected Cancer Based on Visual Inspection, Stratified by Age

Age group, y	No. screened	Colposcopy, No. (%)	Colposcopy compliance rate, %	No.				
				Normal	CIN1	CIN2	CIN3	Cancer
35-39	167	162 (26.2)	97.0	130	28	0	1	0
40-44	202	195 (31.5)	96.5	161	25	4	1	0
45-49	160	154 (24.9)	96.3	128	19	0	1	1
50-54	70	67 (10.8)	95.7	59	4	1	1	1
55-59	30	29 (4.7)	96.7	26	1	0	0	2
60-64	14	12 (1.9)	85.7	9	2	0	0	1
Total	643	619	96.3	513	79	5	4	5

Abbreviations: CIN1, cervical intraepithelial neoplasia grade 1; CIN2, cervical intraepithelial neoplasia grade 2; CIN3, cervical intraepithelial neoplasia grade 3.

## Discussion

### Principal Findings

To our knowledge, this study is the largest population-based screening to evaluate the performance of HPV testing as the primary screening method with visual inspection as a triage method in the same visit. Our findings showed that among HPV-positive women, the overall adherence to triage by visual inspection was 93.9% (22 592 of 24 070), and the colposcopy compliance rate was 65.6% (2714 of 4137). Among HPV-positive women, visual inspection reduced colposcopy referral rates to 18.3% (4137 of 22 592). Detection rates were 2.8% (643 of 22 592) for CIN2+ and 1.9% (438 of 22 592) for CIN3+. The results demonstrated that visual inspection with acetic acid and Lugol iodine can stratify risk among HPV-positive individuals, and the implementation of HPV testing, visual inspection, and colposcopy within 1 visit can effectively mitigate losses to follow-up and detect precancerous lesions. The findings from Ordos City offer valuable insights for areas that face challenges due to limited access to health care resources.

### Performance of Visual Inspection Triage of HPV-Positive Women

Both careHPV and visual inspection were found to be valuable testing methods that are simple, rapid, inexpensive, and well suited for areas with limited health care resources.^19,20,21^ According to a meta-analysis, the careHPV test demonstrated a pooled specificity of 84%,^20^ implying that it may yield false-positive results for 16 of every 100 women without any abnormalities. Our results suggest that visual inspection may serve as an optional triage test among women whose results of the careHPV test were positive. In comparison with a multicenter, randomized clinical trial conducted in rural China in 2015,^22^ visual inspection demonstrated a lower positivity rate (18.3% vs 27.1%), which can be attributed to the subjective diagnosis by different physicians.^12,23^ Meanwhile, a greater detection rate of CIN2+ (2.8% vs 0.5%) and a significant reduction in the number of colposcopy referrals needed to identify 1 case of CIN2+ (4.2 vs 8.0) were observed in our study. These findings highlight the screening efficiency in the practical setting.^22^ Among women who had abnormal results or suspected cancer based on visual inspection, the PPV for CIN2+ detection was 23.7%, which was slightly lower than the PPV in the ESTAMPA (Estudio Multicéntrico de Tamizaje y Triaje de Cáncer de Cuello Uterino con Pruebas del Virus del Papiloma Humano) study (29.9%).^12^ This difference could potentially be associated with the inclusion of the results from an 18-month follow-up in the ESTAMPA study, which accumulated more cases of CIN2+.

When the cohort is stratified by age groups, we refer to the large European population-based screening studies, which offer a more comprehensive categorization of age groups in 5-year intervals.^24,25,26^ Our analysis revealed that triage with visual inspection exhibited a higher detection rate of CIN2+ cases among younger women, which was consistent with previous studies using visual inspection, genotyping, or cytology for HPV-positive triage.^12,27,28^ This phenomenon could be associated with the retreat of the squamocolumnar junction and the resulting challenges in visualizing endocervical disease in postmenopausal women.^29^ This inference is further supported by the median age of 50 years of menopausal women in our study.

### Concurrent Application of HPV Testing and Visual Inspection

An important advantage associated with triage with visual inspection in our study is the possibility of linking screening to immediate management. Reducing visit numbers is crucial in low-resource areas; for each additional visit, approximately one-fourth to one-third of screened women are lost to follow-up.^30,31^ Women in these settings often face economic disadvantages and social barriers and encounter limited access to health care services. Failure to provide adequate care to these women may result in a worse prognosis for screening-detectable lesions.^32^ In our study, the concurrent implementation of HPV testing, visual inspection, and immediate colposcopy yielded an overall adherence rate to triage of 93.9% (22 592 of 24 070) and a colposcopy compliance rate of 69.7% (3333 of 4780).

Despite the overall low rate of colposcopy compliance, the rates of compliance varied based on different visual inspection results. Immediate colposcopy referrals were provided for 686 women with suspected cancer based on visual inspection, with a high compliance rate of 95.0% (652 of 686). Women with abnormal visual inspection results were advised by their health care professional to await the HPV test result to proceed with colposcopy on the same day; the compliance rate for this group was 65.5% (2681 of 4094). These results further underscore the importance of an immediate diagnosis in regions with limited health care resources.

Furthermore, the simultaneous application of both tests during the same visit may reduce the possibility of missed diagnoses of CIN2+ among individuals with a negative HPV result because a negative HPV result does not guarantee a risk-free status.^33^ In our study, we detected a total of 5 cases of cancer among 643 women aged 45 years or older who were HPV negative and received a diagnosis of suspected cancer based on visual inspection. The occurrence of false-negative HPV results can be associated with unsatisfactory sampling among older women, which may result in low sample quality for HPV testing, leading to the inability to detect HPV infection.

### Achievement of the Screening Program in Ordos City

Screening coverage is a key metric for monitoring the performance of the WHO cervical cancer elimination plan. The implementation of a sustainable screening program is crucial to achieving coverage, effectively reducing health disparities, and enhancing health equity in low-resource areas.^34^ Based on our findings, the organized screening program has covered 92.4% of the targeted women and successfully achieved an overall coverage rate of 55.4% among residents within 5 years. The local government retrospectively collected results conducted at different health care facilities in Ordos City in 2022. After removing duplicates, we identified a total of 76 692 women aged 35 to 64 years who underwent opportunistic screening between 2016 and 2020. Our results suggest that a combination of organized screening and opportunistic screening can achieve a 75.5% screening coverage in a 5-year interval in Ordos City.

To our knowledge, this was the largest study that evaluated the the use of HPV testing for primary screening and visual inspection for triage within the same visit in areas with limited health care resources. The extensive sample size used in this study allows for a comprehensive evaluation. Furthermore, the screening program underwent regular supervision by experts from the Chinese Academy of Medical Sciences to ensure the quality of the program. Building on the 5 years of experience, Ordos City initiated a new round of HPV-based cervical cancer screening in 2021, incorporating HPV-16/18 and 12 other genotyping tests.^35^ Besides screening, Ordos City was the first city in China to implement a regional, free HPV vaccination program for girls aged 13 to 18 years starting in 2021.^36^ Ordos City is expected to be the first city in China to eliminate cervical cancer.

### Limitations

Our study was subject to several limitations. The overall compliance rates for colposcopy recalls were not satisfactory. Approximately 30.3% of women (1447 of 4780) refused colposcopy in local hospitals, which could result in an underestimation of the cumulative detection of CIN2+. This hesitation to undergo colposcopy may be because many women with precancer have no symptoms, and because this is the first-ever free cervical cancer screening by Ordos City government for all women aged 35 to 64 years. The program was implemented in a sparsely populated area, where a subset of women was reluctant to follow the advice of health care professionals to stay and await HPV test results. Nonetheless, if a woman opted for a colposcopy or underwent further diagnosis at a different hospital, the woman was advised to bring back the report of the further examination.

## Conclusions

Further investigation is required to explore the cost-effectiveness of the screening approach discussed in this study. Using visual inspection for triage in the same setting in rural China demonstrates higher cost-effectiveness in comparison with cytology for triaging HPV-positive individuals.^37^ Nonetheless, no studies, to our knowledge, have investigated the economic benefits of triaging HPV-positive individuals using visual inspection while conducting both methods during the same visit.

The findings of this cohort study suggest that integrating HPV testing, visual inspection, and colposcopy in a single visit can effectively reduce loss to follow-up, detect precancerous lesions, and prove to be feasible in large-scale population-based cervical cancer screening programs in resource-limited areas of China. These findings hold significant implications for decision-makers in similar health care resource–constrained settings when considering the implementation of cervical cancer screening programs.

## Notes

Supplement.
Data Sharing Statement

## References

[1] Sung H, Ferlay J, Siegel RL, . Global cancer statistics 2020: GLOBOCAN estimates of incidence and mortality worldwide for 36 cancers in 185 countries. CA Cancer J Clin. 2021;71(3):209-249. doi:10.3322/caac.21660 33538338

[2] Han B, Zheng R, Zeng H, . Cancer incidence and mortality in China, 2022. J Natl Cancer Cent. Published online February 2, 2024. doi:10.1016/j.jncc.2024.01.006 PMC11256708 39036382

[3] Gu X, Sun G, Zheng R, et al. Incidence and mortality of cervical cancer in China in 2015. J Natl Cancer Center. 2022;2(2):70-77. doi:10.1016/j.jncc.2022.01.002 PMC11256676 39034952

[4] Global strategy to accelerate the elimination of cervical cancer as a public health problem. World Health Organization. November 17, 2020. Accessed June 27, 2023. https://www.who.int/publications/i/item/9789240014107

[5] Lin S, Gao K, Gu S, . Worldwide trends in cervical cancer incidence and mortality, with predictions for the next 15 years. Cancer. 2021;127(21):4030-4039. doi:10.1002/cncr.33795 34368955

[6] Altobelli E, Rapacchietta L, Profeta VF, Fagnano R. HPV-vaccination and cancer cervical screening in 53 WHO European countries: an update on prevention programs according to income level. Cancer Med. 2019;8(5):2524-2534. doi:10.1002/cam4.2048 30993902 PMC6536990

[7] Sankaranarayanan R. Screening for cancer in low- and middle-income countries. Ann Glob Health. 2014;80(5):412-417. doi:10.1016/j.aogh.2014.09.014 25512156

[8] Denny L. Cytological screening for cervical cancer prevention. Best Pract Res Clin Obstet Gynaecol. 2012;26(2):189-196. doi:10.1016/j.bpobgyn.2011.08.001 22071306

[9] WHO guideline for screening and treatment of cervical pre-cancer lesions for cervical cancer prevention: second edition. World Health Organization. July 6, 2021. Accessed July 26, 2023. https://www.who.int/publications/i/item/9789240030824 34314129

[10] Magdi R, Elshafeey F, Elshebiny M, ; ECEBM Working Group. A systematic review and meta-analysis of diagnostic accuracy of HPV tests for the screening of cervical cancer in low-resource settings. Int J Gynaecol Obstet. 2021;152(1):12-18. doi:10.1002/ijgo.13455 33124048

[11] Wentzensen N, Schiffman M, Palmer T, Arbyn M. Triage of HPV positive women in cervical cancer screening. J Clin Virol. 2016;76(suppl 1):S49-S55. doi:10.1016/j.jcv.2015.11.015 26643050 PMC4789103

[12] Baena A, Mesher D, Salgado Y, ; ESTAMPA study group. Performance of visual inspection of the cervix with acetic acid (VIA) for triage of HPV screen-positive women: results from the ESTAMPA study. Int J Cancer. 2023;152(8):1581-1592. doi:10.1002/ijc.34384 36451311 PMC10107773

[13] Dang L, Kong L, Zhao Y, . Evaluation of triage strategies for high-risk human papillomavirus–positive women in cervical cancer screening: a multicenter randomized controlled trial in different resource settings in China. Chin J Cancer Res. 2022;34(5):496-509. doi:10.21147/j.issn.1000-9604.2022.05.09 36398123 PMC9646459

[14] Di J, Rutherford S, Chu C. Review of the cervical cancer burden and population-based cervical cancer screening in China. Asian Pac J Cancer Prev. 2015;16(17):7401-7407. doi:10.7314/APJCP.2015.16.17.7401 26625735

[15] Zhang M, Zhong Y, Wang L, . Cervical cancer screening coverage—China, 2018–2019. China CDC Wkly. 2022;4(48):1077-1082. doi:10.46234/ccdcw2022.217 36751373 PMC9889230

[16] Fu Y, Wang J, Sun J, Zhang S, Huang D. Equity in the allocation of general practitioner resources in mainland China from 2012 to 2019. Healthcare (Basel). 2023;11(3):398. doi:10.3390/healthcare11030398 36766973 PMC9913937

[17] Data of Ordos City. Ordos City. Accessed June 9, 2023. http://sj.tjj.ordos.gov.cn/datashow/index.htm

[18] WHO prequalification of in vitro diagnostics: public report: product: careHPV test. World Health Organization. July 2018. Accessed June 7, 2023. https://extranet.who.int/prequal/sites/default/files/whopr_files/PQDx_0085-028-00_CareHPVTest_v1.pdf

[19] Catarino R, Schäfer S, Vassilakos P, Petignat P, Arbyn M. Accuracy of combinations of visual inspection using acetic acid or Lugol iodine to detect cervical precancer: a meta-analysis. BJOG. 2018;125(5):545-553. doi:10.1111/1471-0528.14783 28603909

[20] Kelly H, Mayaud P, Segondy M, Pant Pai N, Peeling RW. A systematic review and meta-analysis of studies evaluating the performance of point-of-care tests for human papillomavirus screening. Sex Transm Infect. 2017;93(S4):S36-S45. doi:10.1136/sextrans-2016-053070 29223961

[21] Jeronimo J, Bansil P, Lim J, ; START-UP Study Group. A multicountry evaluation of careHPV testing, visual inspection with acetic acid, and papanicolaou testing for the detection of cervical cancer. Int J Gynecol Cancer. 2014;24(3):576-585. doi:10.1097/IGC.0000000000000084 24557438 PMC4047307

[22] Zhang J, Zhao Y, Dai Y, . Effectiveness of high-risk human papillomavirus testing for cervical cancer screening in China: a multicenter, open-label, randomized clinical trial. JAMA Oncol. 2021;7(2):263-270. doi:10.1001/jamaoncol.2020.6575 33377903 PMC7774051

[23] Asgary R, Beideck E, Naderi R. Comparative assessment of test characteristics of cervical cancer screening methods for implementation in low-resource settings. Prev Med. 2022;154:106883. doi:10.1016/j.ypmed.2021.106883 34785209

[24] Veldhuijzen NJ, Berkhof J, Gillio-Tos A, . The age distribution of type-specific high-risk human papillomavirus incidence in two population-based screening trials. Cancer Epidemiol Biomarkers Prev. 2015;24(1):111-118. doi:10.1158/1055-9965.EPI-14-0628 25300476

[25] Leinonen MK, Anttila A, Malila N, Dillner J, Forslund O, Nieminen P. Type- and age-specific distribution of human papillomavirus in women attending cervical cancer screening in Finland. Br J Cancer. 2013;109(11):2941-2950. doi:10.1038/bjc.2013.647 24136148 PMC3844908

[26] Vänskä S, Luostarinen T, Lagheden C, . Differing age-specific cervical cancer incidence between different types of human papillomavirus: implications for predicting the impact of elimination programs. Am J Epidemiol. 2021;190(4):506-514. doi:10.1093/aje/kwaa121 32639531 PMC8024050

[27] Rebolj M, Mathews CS, Pesola F, Cuschieri K, Denton K, Kitchener H; HPV Pilot Steering Group. Age-specific outcomes from the first round of HPV screening in unvaccinated women: observational study from the English cervical screening pilot. BJOG. 2022;129(8):1278-1288. doi:10.1111/1471-0528.17058 34913243

[28] Gori S, Battagello J, Gustinucci D, . Clinical relevance of partial HPV16/18 genotyping in stratifying HPV-positive women attending routine cervical cancer screening: a population-based cohort study. BJOG. 2021;128(8):1353-1362. doi:10.1111/1471-0528.16631 33326680 PMC8248328

[29] Holt HK, Zhang L, Zhao FH, . Evaluation of multiple primary and combination screening strategies in postmenopausal women for detection of cervical cancer in China. Int J Cancer. 2017;140(3):544-554. doi:10.1002/ijc.30468 27727464 PMC13034481

[30] Garcia A, Juarez M, Sacuj N, . Loss to follow-up and the care cascade for cervical cancer care in rural Guatemala: a cross-sectional study. JCO Glob Oncol. 2022;8:e2100286. doi:10.1200/GO.21.00286 35113733 PMC8853617

[31] Kiptoo S, Otieno G, Tonui P, . Loss to follow-up in a cervical cancer screening and treatment program in western Kenya. J Global Oncol*.* 2018;4(suppl 2):97s. doi:10.1200/jgo.18.41300

[32] Jørgensen SF, Andersen B, Rebolj M, Njor SH. Gaps between recommendations and their implementation: a register-based study of follow-up after abnormalities in cervical cancer screening. Prev Med. 2021;146:106468. doi:10.1016/j.ypmed.2021.106468 33636193

[33] Petry KU, Cox JT, Johnson K, . Evaluating HPV-negative CIN2+ in the ATHENA trial. Int J Cancer. 2016;138(12):2932-2939. doi:10.1002/ijc.30032 26851121 PMC5069615

[34] Campos NG, Tsu V, Jeronimo J, Mvundura M, Lee K, Kim JJ. To expand coverage, or increase frequency: quantifying the tradeoffs between equity and efficiency facing cervical cancer screening programs in low-resource settings. Int J Cancer. 2017;140(6):1293-1305. doi:10.1002/ijc.30551 27925175 PMC5516173

[35] Implementing “Two Cancers” prevention and treatment program to protect women’s health 2021. Ordos Municipal Health Commission. Accessed August 24, 2023. http://wjw.ordos.gov.cn/zdzt/jkeedsxd/202109/t20210922_2999270.html

[36] China’s first free HPV vaccine starts in Ordos Junggar Banner. Ordos Government. 2020. Accessed July 8, 2023. https://www.ordos.gov.cn/gk_128120/wsjkly/zccs_2/202008/t20200818_2735841.html

[37] Wang YY, Wang ZJ, Zhang Y, . Economic evaluation of fifteen cervical cancer screening strategies in rural China. Article in Chinese. Zhonghua Fu Chan Ke Za Zhi. 2019;54(12):840-847. 31874474 10.3760/cma.j.issn.0529-567x.2019.12.008

