# Supplementary material for: Cervical Cancer Screening via Visual Inspection With Acetic Acid and Lugol Iodine for Triage of HPV-Positive Women
Source: JAMA Netw Open. 2024 Mar 29;7(3):e244090. doi: 10.1001/jamanetworkopen.2024.4090 (PMC10980959; doi:10.1001/jamanetworkopen.2024.4090)
Supplement: Supplement. — Data Sharing Statement [file jamanetwopen-e244090-s001.pdf]

Data Sharing Statement

Wang. Cervical Cancer Screening via Visual Inspection With Acetic Acid and Lugol Iodine for Triage of HPV-Positive Women. *JAMA Netw Open*. Published March 29, 2024. doi:10.1001/jamanetworkopen.2024.4090

Data
Data available: No
